# Demystifying the oncologic patient in the intensive care unit

**DOI:** 10.62675/2965-2774.20240054-en

**Published:** 2024-10-07

**Authors:** Antonio Paulo Nassar, Laura Inez de Oliveira Santos

**Affiliations:** 1 Intensive Care Unit A. C. Camargo Cancer Center São Paulo SP Brazil Intensive Care Unit, A. C. Camargo Cancer Center - São Paulo (SP), Brazil.

Advances in cancer diagnosis and treatment have led to an increase in the number of patients with cancer admitted to the intensive care unit (ICU) in recent years.^( 1 )^ These developments underscore the transformation of critically ill patients with cancer, once deemed terminal, into a regular facet of intensivists’ caseloads, emphasizing the need for closer collaboration between intensivists and oncologists to formulate evidence-based therapeutic strategies. Thus, intensivists must grasp the nuances of various cancers, their complications, and patient support requirements to deliver tailored, high-quality care.^( 2 )^

The cumulative incidence of ICU admissions of patients with hematological and solid malignancies is higher in the first year after diagnosis, possibly attributable to the aggressiveness of initial treatment regimens, clinical complications, and exigencies of postoperative care.^( 3 , 4 )^ Consequently, the adoption of “high-value care”, encompassing early admission to the ICU, comprehensive etiological investigation of decompensation, and the implementation of therapeutic proportionality, among other measures, is imperative. This approach aims to reduce mortality rates and improve short- and long-term results.^( 2 )^

However, prevailing prejudices surrounding the care of critically ill patients with cancer pose potential hindrances to their treatment. Demystifying the notion that ICU admission for such patients equates to a terminal outcome is paramount. Numerous studies have reported reduced mortality rates, even among patients requiring advanced life support, such as dialysis, vasopressor, and mechanical ventilation. A meta-analysis of individual patient data revealed a 4% annual decline in mortality rates between 1995 and 2010.^( 5 )^Moreover, a Brazilian multicenter study involving over 30,000 patients demonstrated a cumulative 10% reduction in hospital mortality within a decade.^( 1 )^ While acute organ dysfunctions, particularly the requiring mechanical ventilation, are acknowledged as significant short-term mortality indicators, preexisting functional impairment has emerged as the main risk factor among patients with less severe acute dysfunctions.^( 6 , 7 )^ Nevertheless, acute organ dysfunctions also have adverse effects on postdischarge survival rates, exemplified by higher incidences of treatment alterations or suspensions, as evidenced among patients experiencing delirium during their ICU stay.^( 8 )^ Hence, considerations of preexisting functional impairment, acute dysfunction severity, and progression are pivotal in decision-making concerning the care of critically ill patients with cancer.

Long-term outcomes are primarily defined by the response to cancer treatment.^( 9 )^ Studies have reported lower mortality rates among critically ill patients with solid tumors than among those with hematological malignancies, with notably poorer outcomes among recipients of allogeneic bone marrow transplants.^( 5 )^Noteworthy advancements in contemporary treatments, such as targeted therapy, immune checkpoint inhibitors, and chimeric antigen receptor (CAR) T-cell therapy, have improved the long-term prognosis of patients with advanced and/or metastatic cancer, allowing even elderly patients with multiple comorbidities to maintain an acceptable quality of life.^( 10 )^

Recognizing differential tumor responses to treatment is pivotal in determining ICU admission and the provision of artificial life support. For example, patients with non-small cell lung cancer with an epidermal growth factor receptor (EGFR) mutation exhibit enhanced responses to targeted therapy even at locally advanced stages.^( 11 )^ Distinct molecular markers and corresponding therapies have also been associated with improved long-term survival in patients with breast cancer, colon cancer, melanoma, and other cancers. Improvements in the treatment of hematological malignancies such as multiple myeloma have likewise contributed to increased likelihood of survival and improved quality of life.^( 12 )^ However, practicing precision medicine in intensive care medicine remains elusive while representing a long-awaited step that could increase survival rates among critically ill patients with and without cancer.

Given the intricate nature of diverse types of cancer and their molecular profiles, the scope surpasses intensivists’ expertise. Consequently, the sharing of perceptions through daily interdisciplinary meetings between oncologists and intensivists appears beneficial, promoting better patient care, reducing hospital mortality rates, and optimizing the use of resources.^( 13 )^ However, inherent disparities in focus often lead to conflicts between teams; while oncologists prioritize survival rates on the basis of the type of cancer for decision-making, intensivists focus on survival rates that depend on multiple organ dysfunction.^( 14 )^Merging these two fields of expertise undoubtedly enhances the care of critically ill patients with cancer.

Regrettably, despite recent improvements in cancer treatment, many patients with advanced cancer, such as those with poor performance statuses, leptomeningeal and peritoneal carcinomatosis, malignant hypercalcemia, and obstructive jaundice, are unlikely to benefit from aggressive critical care interventions. Consequently, intensivists and oncologists caring for such patients must assess patients’ understanding of the disease, hopes, concerns, and values to formulate suitable treatment plans.^( 15 )^

Thus, recent progress in the treatment of cancer has made it a more manageable chronic disease for the majority of patients. Intensivists must remain aware of how these advancements reshape their daily practices, partnering with oncologists and hematologists and continuing to provide exemplary, dedicated, compassionate, and evidence-based care to critically ill patients.
