# Supplementary material for: Demystifying the oncologic patient in the intensive care unit
Source: Crit Care Sci. 2024 Oct 7;36:e20240054en. doi: 10.62675/2965-2774.20240054-en (PMC11463986; doi:10.62675/2965-2774.20240054-en)
Supplement: Translation

# Translation: Desmistificando o paciente oncológico na unidade de terapia intensiva

**DOI:** 10.62675/2965-2774.20240054-pt

**Authors:** Antonio Paulo Nassar, Laura Inez de Oliveira Santos

**Affiliations:** 1 Brasil Unidade de Terapia Intensiva, A. C. Camargo Cancer Center - São Paulo (SP), Brasil.

Os avanços no diagnóstico e no tratamento do câncer aumentaram o número de pacientes com câncer internados na unidade de terapia intensiva (UTI) nos últimos anos.^( 1 )^Esses avanços ressaltam a transformação de pacientes críticos com câncer, antes considerados terminais, em uma faceta normal dos casos dos intensivistas, enfatizando a necessidade de uma colaboração mais próxima entre intensivistas e oncologistas para formular estratégias terapêuticas baseadas em evidências. Assim, os intensivistas devem compreender as nuances de vários tipos de câncer, suas complicações e os requisitos de suporte ao paciente, para prestar assistência personalizada e de alta qualidade.^( 2 )^

A incidência cumulativa de internações na UTI de pacientes com neoplasias malignas hematológicas e sólidas é maior no primeiro ano após o diagnóstico, possivelmente atribuível à agressividade dos esquemas de tratamento inicial, às complicações clínicas e às exigências dos cuidados pós-operatórios.^( 3 , 4 )^Consequentemente, é imprescindível a adoção de “cuidados de alto valor”, abrangendo a admissão precoce na UTI, a investigação etiológica abrangente da descompensação e a implementação da proporcionalidade terapêutica, entre outras medidas. Essa abordagem visa reduzir as taxas de mortalidade e melhorar os resultados a curto e longo prazo.^( 2 )^

No entanto, preconceitos predominantes em torno da assistência a pacientes críticos com câncer representam possíveis obstáculos ao seu tratamento. Torna-se fundamental desmistificar a noção de que a admissão desses pacientes na UTI equivale a um desfecho terminal. Diversos estudos relataram taxas de mortalidade reduzidas, mesmo entre pacientes que necessitam de suporte avançado de vida, como diálise, vasopressor e ventilação mecânica. Uma metanálise de dados de pacientes individuais revelou declínio anual de 4% nas taxas de mortalidade entre 1995 e 2010.^( 5 )^Além disso, um estudo multicêntrico brasileiro envolvendo mais de 30 mil pacientes demonstrou uma redução cumulativa de 10% na mortalidade hospitalar em uma década.^( 1 )^Embora as disfunções orgânicas agudas, particularmente aquelas que requerem ventilação mecânica, sejam reconhecidas como indicadores significativos de mortalidade em curto prazo, o comprometimento funcional preexistente surgiu como o principal fator de risco entre os pacientes com disfunções agudas menos graves.^( 6 , 7 )^No entanto, as disfunções orgânicas agudas também têm efeitos adversos sobre as taxas de sobrevida após a alta, exemplificadas por incidências mais altas de alterações ou suspensões de tratamento, conforme evidenciado entre os pacientes que apresentaram *delirium* durante a internação na UTI.^( 8 )^Portanto, as considerações sobre o comprometimento funcional preexistente, a gravidade e a progressão da disfunção aguda são fundamentais para a tomada de decisões relativas ao tratamento de pacientes críticos com câncer.

Os desfechos duradouros são definidos principalmente pela resposta ao tratamento do câncer.^( 9 )^Estudos relataram taxas de mortalidade mais baixas entre pacientes críticos com tumores sólidos do que entre aqueles com malignidades hematológicas, com desfechos notavelmente menos favoráveis entre os receptores de transplantes alogênicos de medula óssea.^( 5 )^Avanços notáveis nos tratamentos contemporâneos, como terapia direcionada, inibidores dos pontos de verificação imunológicos e terapia com células T de receptor de antígeno quimérico (CAR - *chimeric antigen receptor* ), melhoraram o prognóstico em longo prazo de pacientes com câncer avançado e/ou metastático, permitindo que até mesmo pacientes idosos com múltiplas comorbidades mantenham uma qualidade de vida aceitável.^( 10 )^

O reconhecimento das respostas diferenciais do tumor ao tratamento é fundamental para determinar a admissão na UTI e o fornecimento de suporte artificial à vida. Por exemplo, pacientes com câncer de pulmão de células não pequenas com uma mutação no receptor do fator de crescimento epidérmico (EGFR - *epidermal growth factor receptor* ) apresentam melhores respostas à terapia direcionada, mesmo em estágios localmente avançados.^( 11 )^Marcadores moleculares distintos e terapias correspondentes também foram associados a uma melhor sobrevida prolongada em pacientes com câncer de mama, câncer de cólon, melanoma e outros tipos de câncer. Avanços no tratamento de malignidades hematológicas, como o mieloma múltiplo, também contribuíram para aumentar a probabilidade de sobrevida e melhorar a qualidade de vida.^( 12 )^No entanto, a prática da medicina de precisão na medicina de terapia intensiva ainda é ilusória, embora represente uma etapa há muito esperada, que poderia aumentar as taxas de sobrevida entre pacientes críticos com e sem câncer.

Dada a natureza intrincada de diversos tipos de câncer e seus perfis moleculares, o escopo ultrapassa a experiência dos intensivistas. Consequentemente, o compartilhamento de percepções por meio de reuniões interdisciplinares diárias entre oncologistas e intensivistas parece ser benéfico, promovendo melhor assistência ao paciente, reduzindo as taxas de mortalidade hospitalar e otimizando o uso de recursos.^( 13 )^No entanto, as disparidades inerentes ao foco muitas vezes levam a conflitos entre as equipes; enquanto os oncologistas priorizam as taxas de sobrevida conforme o tipo de câncer para a tomada de decisões, os intensivistas se concentram nas taxas de sobrevida que dependem da disfunção de múltiplos órgãos.^( 14 )^A fusão desses dois campos de especialização, sem dúvida, aperfeiçoa a assistência a pacientes críticos com câncer.

Lamentavelmente, apesar das recentes conquistas no tratamento do câncer, muitos pacientes com câncer avançado, como aqueles com baixo nível de desempenho, carcinomatose leptomeníngea e peritoneal, hipercalcemia maligna e icterícia obstrutiva, provavelmente não se beneficiarão de intervenções agressivas de tratamento intensivo. Consequentemente, os intensivistas e os oncologistas que assistem a esses pacientes devem avaliar a compreensão da doença, as expectativas, as preocupações e os valores dos pacientes para formular planos de tratamento adequados.^( 15 )^

Assim, o recente avanço no tratamento do câncer tornou-o uma doença crônica mais controlável para a maioria dos pacientes. Os intensivistas devem ter consciência de como esses avanços remodelam suas práticas diárias, fazendo parcerias com oncologistas e hematologistas e continuando a prestar assistência exemplar, dedicada, compassiva e baseada em evidências a pacientes críticos.
